# Synthesis of N-Benzylideneaniline by Schiff base reaction using Kinnow peel powder as Green catalyst and comparative study of derivatives through ANOVA techniques

**DOI:** 10.1038/s41598-022-13360-5

**Published:** 2022-06-10

**Authors:** Renu Verma, Narendra Pal Lamba, Anshu Dandia, Anamika Srivastava, Kanak Modi, Manmohan Singh Chauhan, Jagdish Prasad

**Affiliations:** 1 ASAS, Amity University Rajasthan, Jaipur, Rajasthan 303002 India; 2 Department of Chemistry, University of Rajasthan, Jaipur, 302004 India; 3 Department of Chemistry, Banssthali Vidyapith, Banasthali, 304022 India

**Keywords:** Chemistry, Mathematics and computing

## Abstract

The cheap and easy availability of the Kinnow peel waste has reported various applications due to presence of multifunctional groups. Therefore, in present study we explored its application to synthesize N-Benzylideneaniline and its derivatives based on Schiff base reaction. Kinnow peel powder is characterized by FTIR, TEM, SEM, XRD, EDX, and TGA for functional groups, morphology, surface, elements and thermal stability. Benzaldehyde, aniline, and their derivatives such as 4-methyl benzaldehyde, 4-hydroxy benzaldehyde, 4-methoxy benzaldehyde, and 4-methoxy aniline have been used to compare the efficacy of the Schiff base reaction using analysis of variance (ANOVA) and it has been observed that combination of Aniline and benzaldehyde for Schiff base reaction provided 85% yield of relative product.

## Introduction

In the present time, it becomes a new challenge to reduce chemical waste, reaction time, economically efficient and toxic chemicals in organic transformations for the researchers. Green chemistry plays the most fundamental role in synthetic chemistry because it maximize the yield of reaction product and minimize the side product without using any hazardous chemicals and specific reaction conditions, so it become possible to minimize the harassment of environment. Green catalyst is only the path, which can resolve these all issues. There are many green catalysts and green methods are reported to make environment toxic free like modified of mesoporous halloysite nanotubes (HNTs) by using CuFe_2_O_4_ nanopartical^1^, synthesized the imidazole heterocycles via green NiFe_2_O_4_/geopolymer nanocatalyst^2^, and preparation of nickel nanoparticles by green method^3^. Green catalysts are also used in synthesis of 2,4,6-tri-arylpyridines, diazepine, organic transformation, pyrido-imidazo-isoquinolines, synthesis of chromene-linked nicotinonitriles, and aminonitriles by using the environmentally-friendly and highly efficient LPSF magnetic^4^, Fe_3_O_4_/SiO_2_^5^, iron oxide nanoparticles^6^, multiple carbon nanotubes and TiO_2_ catalyst^7^, Fe_3_O_4_@SiO_2_-OSO_3_H^8^, nanobiocomposite^9^, and Fe_3_O_4_@SiO_2_-NH_2_-GA^10^ nanocatalyst are reported respectively. Cellulose based nanocomposite with Fe_3_O_4_ nanoparticles is reported as a recoverable catalyst for condensation reaction^11^. A novel and green nanocomposite Fe_3_O_4_@PEO-SO_3_H, (PEG-400)-SO_3_H-coated Fe_2_O_3_ and copper oxide nanoparticles were prepared for green synthesis of polyhydroquinolines^12^, aminocarbonyl^13^, and 1, 2,3-triazoles ^14^ respectively.

Biomass is a cheap carbon material, so, it is important and abundant in nature^15^. Biomass is generally derived from mesoporous carbon, which has great potential because of its chemical, mechanical and physical properties^16,17^. Hence, biomass have wide applications in the field of sensors^18–20^, catalysis^21–25^, gas storage^26–28^, energy storage^29–34^, and waste-water treatment^35,36^. Biomass contains many oxygen functional group and other elements at their surface, which makes it a good support material for catalyst^37^. However, many biomass-based catalysts were reported and obtained from fruits peel and were found to be inefficient for catalytic purposes^38–40^ such as catalyst developed from leftover pulp of *Citrus limetta* (Mausambi) which was waste material^41,42^. Therefore, it is necessary to develop novel biomass catalyst that will be more stable and reliable for many applications.

Hugo Schiff reported the Schiff base by condensation reaction between aldehyde and amine in 1864^43^. These compounds contains azomethine group (–HC=N–) and behaves like Flexi-dentate ligand. Schiff base have wide applications in many types of biological activities like antibacterial^44^, antitumor^45^, anti-oxidant^46^, anti-inflammatory activity^47^, antifungal^48^ and industrial applications. Schiff base ligand is generally used in the development of inorganic chemistry, co-ordination chemistry because they are able to generate complex with metal ions. Some of the Schiff base shows good catalytic performance at high temperature^49^. Synthesis of Schiff base is catalyzed by various type of chemical catalyst. However, these catalysts may be harmful for environment as well as for human being. To overcome these issues, some eco-friendly catalyst developed and reported.

Due to the high importance of eco-friendly catalysis in the Schiff Base reaction and potential of cost effectively, available fruit peel experiments have been designed to investigate its application as a catalyst in the present study. This is very first report where Kinnow peel powder is used as organic catalyst for Schiff base reaction between aniline and benzaldehyde.

## Experimental methodology

### Materials

Waste peels of Kinnow mandarin is selected and used as a catalyst in this study. Kinnow peels are collected from local fruit stalls in Jaipur, Rajasthan. Bruker Fourier transform infrared spectroscopy (FT-IR), Scanning electron microscopy (SEM), Thermogravimetric analysis (TGA), Energy dispersion X-ray spectroscopy (EDS), X-ray diffraction (XRD), Transmission emission spectroscopy (TEM), and Thin layer chromatography (TLC) are performed for characterization of Kinnow peel powder. All chemicals and solvents are used without any purification: DMSO, DCM, acetonitrile, pet-ether, diethyl ether, aniline, benzaldehyde, 95% ethanol, and distilled water.

### Preparation of the catalyst

**Figure 1:**
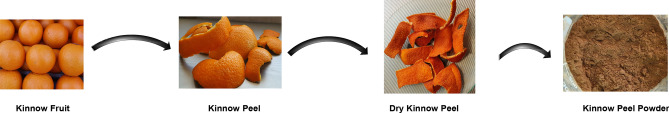
Schematic representation of preparation of the catalyst (Kinnow Peel powder).

Kinnow peel wastes are washed with di-ionized water to remove dirt particles and cleaned well. The peel is dried in an oven for 24 h at 70 ºC to remove moisture content. The dried peel waste is converted into powder form and then the powder is stored for further experiment (Fig. 1).

### Preparation of Schiff base and their derivatives

10 mg of Kinnow peel powder is added in reaction mixture of 1 mmol of Benzaldehyde and 1 mmol of Aniline in a test tube and allowed to stir it for 3 min on the magnetic stirrer at room temperature. The performance of the reaction is checked by the TLC plates with mobile phase [9:1 ratio of hexane and ethyl acetate] and after the completion of catalytic reaction the desired product is recrystallized by ethanol. Similar method is used for Schiff base reaction between derivatives of aniline and benzaldehyde.

## Results and discussion

**Scheme 1:**
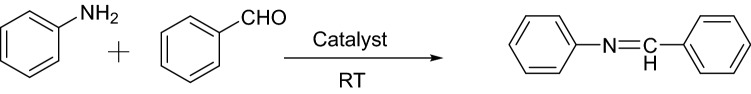
Schiff base reaction between benzaldehyde and aniline.

Herein, synthesis and to obtained high yield of the Schiff base product with green catalyst (organic material) is developed. This is achieved by adopting the green method for Schiff base reaction between benzaldehyde and aniline with Kinnow peel powder. It is observed that the desired product N-Benzylideneaniline is formed with 85% yield in 3 min (Scheme 1).

### Optimization for Schiff base reaction:

The Schiff base reaction in between benzaldehyde and aniline is performed with various solvents (Table 1). From Table 1 it is clear that, 72% yield of relative product is observed with DCM (Table 1, entry 1), while DMSO provides only 70% yield (Table 1, entry 2). We also used diethyl ether, pet ether, acetonitrile for Schiff base reaction and 65–75% yield of relative products was found respectively (Table 1, entry 3–5). We also investigated the effect of catalyst loading, it is observed that 10 mg catalyst provides best results (Table 1, entry 7) but without catalyst Schiff base reaction provides only 48% yield of relative product (Table 1, entry 9). The bare component also provides moderate yield (60%) of Schiff base product (Table 1, entry 10).

**Table 1:** The effect of solvents and different amount of catalyst on Schiff base reaction between aniline and benzaldehyde.

Entry	Solvents	Catalyst (mg)	Time (min)	Yield (%)
1	DCM	10	180	72
2	DMSO	10	240	70
3	Diethyl ether	10	210	65
4	Pet ether	10	150	68
5	Acetonitrile	10	240	75
6	Neat	5	5	75
7	Neat	10	3	85
8	Neat	20	3	85
9	Neat	Without catalyst	10	48
10	Neat	Bare component	10	60

Therefore, Kinnow peel powder is high stable and eco-friendly catalyst for synthesis of Schiff base and providing best results under mild reaction conditions.

**Scheme 2:**
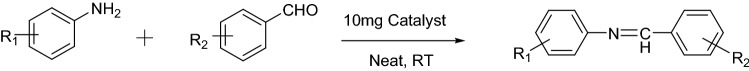
Schiff base reaction between benzaldehyde and aniline.

Derivative of N-Benzylideneaniline also synthesized using same experimental procedure. The different derivatives such as 4-methyl benzaldehyde, 4- hydroxy benzaldehye, 4-methoxy benzaldehyde, and 4-methoxy aniline are used for Schiff base reaction by following optimized reaction conditions. Further more, it has been also observed that derivatives of aniline and benzaldehyde also affect the yield of relative Schiff base products. After that to confirm the yield of respective reaction, each derivative reaction of Schiff base put on five times and observed the result (As shown in Table 2) (Scheme 2).

**Table 2:** Schiff base reaction by using different derivatives of aniline and benzaldehyde.

Entry	Aromatic amine	Aldehyde derivatives	Product	%Yield in consecutive 5 cycles
1				85, 85,84, 83, 85
2				80, 80,80, 79, 78
3				80, 80, 78, 80, 80
4				82, 80, 82, 83, 83
5				83, 83, 84, 83, 83
6				80, 80, 81, 80, 80

The total number of sampled observation of the yield of six derivatives are not the same, therefore the difference among the effect of the derivatives to obtain by considering the following hypothesis.

The null hypothesis (H_0_): is that there is no difference among the effect of six derivatives on the yield of the product against the alternative hypothesis (H_1)_: is that there is a difference among the effect of six derivatives on the yield of the product.

To test the above null hypothesis one-way analysis of variance (ANOVA) technique is used. Before applying the ANOVA technique, the assumption for homogeneity of variances is tested through Levene’s test. For analysis of the data the software SPSS is used. From SPSS following Descriptive statistics is obtained (Table 3).

**Table 3:** Descriptive statistics of the product of Schiff base reaction using various substrates catalyzed by Kinnow peel powder.

	N	Mean	Std. deviation	Std. error	95% Confidence interval for mean		Minimum	Maximum
					Lower bound	Upper bound		
Aniline + Benzaldehyde	5	84.4000	.89443	.40000	83.2894	85.5106	83.00	85.00
Aniline + 4methyl Benzaldehyde	5	79.4000	.89443	.40000	78.2894	80.5106	78.00	80.00
Aniline + 4-hydroxy Benzaldehyde	5	79.6000	.89443	.40000	78.4894	80.7106	78.00	80.00
Aniline + 4 Methoxy Benzaldehyde	5	82.0000	1.22474	.54772	80.4793	83.5207	80.00	83.00
4-Methoxy Aniline + Benzaldehyde	5	83.2000	.44721	.20000	82.6447	83.7553	83.00	84.00
4-Methoxy Aniline + 4-Methyl Benzaldehyde	5	80.2000	.44721	.20000	79.6447	80.7553	80.00	81.00
Total	30	81.4667	2.06336	.37672	80.6962	82.2371	78.00	85.00

The analysis of variance table for testing the difference among the derivatives on the yield is given below (Table 4).

**Table 4:** Analysis of Variance (ANOVA) table presenting statistical difference between the treatments (Combination of the reactants).

	Sum of squares	Df	Mean square	F	Sig
Between Groups	106.267	5	21.253	29.656	.000
Within Groups	17.200	24	.717		
Total	123.467	29			

From the above table, it is concluded that there is a difference of the yield among the six derivatives. Since, there is a difference among six derivatives then a multiple comparison test that is a Tukey test is applied to find out the significant difference between any two derivatives.

From the Table 5 it is concluded that Aniline + Benzaldehyde has significant difference among these four derivatives, Aniline + 4-methyl benzaldehyde, Aniline + 4-hydroxybenzaldehyde, Aniline + 4-methoxy benzaldehyde, and 4-methoxy aniline + 4-methyl benzaldehyde. Aniline + benzaldehyde is not significantly different with 4-methoxy aniline + benzaldehyde. So on the basis of descriptive statistics the average yield of the product from the derivative Aniline + Benzaldehyde is more than 4-methoxy aniline + benzaldehyde. Therefore, the Aniline + Benzaldehyde has significant effect on the yield of the product.

**Table 5:** Homogeneity test results of Tukey HSD for the yield of Schiff base reaction.

	(I) Substrate	(J) Substrate	Mean difference (I-J)	Std. error	Sig	95% confidence interval	
						Lower bound	Upper bound
Tukey HSD	Aniline + Benzaldehyde	Aniline + 4methyl Benzaldehyde	5.00000*	.53541	.000	3.3445	6.6555
		Aniline + 4-hydroxy Benzaldehyde	4.80000*	.53541	.000	3.1445	6.4555
		Aniline + 4 Methoxy Benzaldehyde	2.40000*	.53541	.002	.7445	4.0555
		4 Methoxy Aniline + Benzaldehyde	1.20000	.53541	.256	-.4555	2.8555
		4 Methoxy Aniline + 4 Mtheyl Benzaldehyde	4.20000*	.53541	.000	2.5445	5.8555

### IR spectrum of fresh and recovered Kinnow peel powder

**Figure 2:**
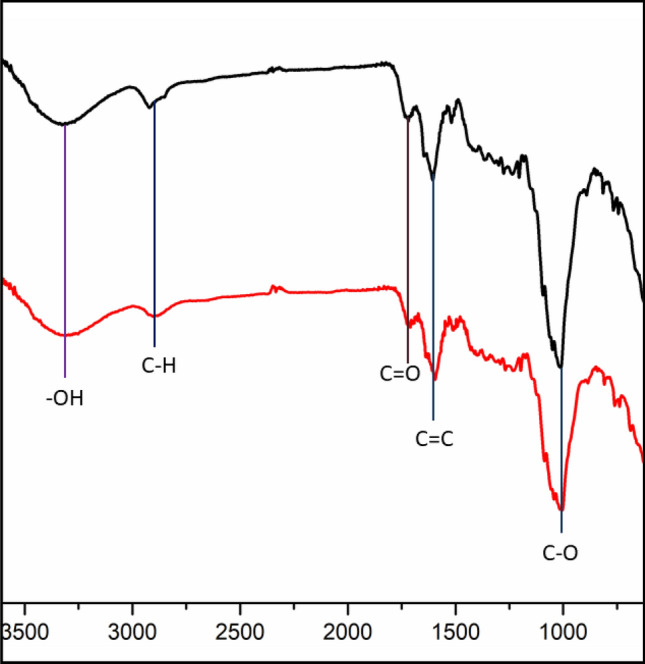
IR spectrum of fresh Kinnow peel powder (black) and recovered Kinnow peel powder (red).

IR spectrum of fresh Kinnow peel powder and recovered Kinnow peel powder is compared (Fig. 2), which shows a broad peak at 3325 cm^-1^ and 3328 cm^-1^ in the high-frequency area attributed to the stretching mode of the O–H bond, which reveals the presence of hydroxyl groups in both Kinnow peel powder. The C-H stretching observed at 2920 cm^-1^ and 2918 cm^-1^. The bands observed at 1718 cm^-1^ and 1720 cm^-1^ are assigned to the carboxyl group. The sharp peak found at 1605 cm^-1^ and 1603 cm^-1^ is a resonance peak which assigned to C = C (aromatic ring). The peak at 1419 cm^-1^ and 1406 cm^-1^ denotes COO^-^ bond and the peak at 1093 cm^-1^ and 1094 cm^-1^ corresponds to the vibrational mode of the C-O group.

### TEM

**Figure 3:**
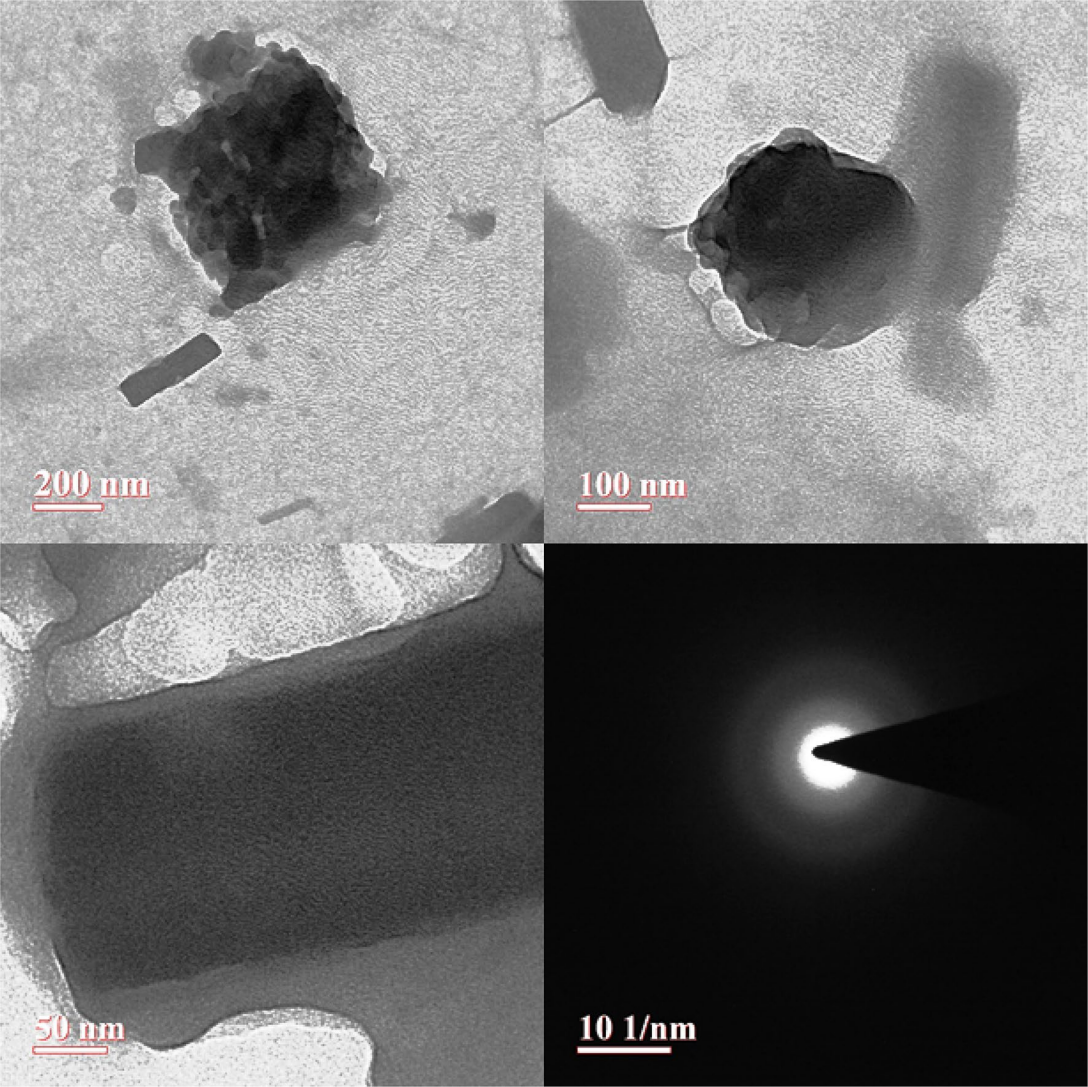
TEM analysis of Kinnow peel powder.

The TEM is used to determine the morphology of Kinnow peel powder. TEM images confirmed that the samples' particles are spherical having diameter of > 100 nm and rod shaped with > 50 nm in length (Fig. 3) and that the particles are largely agglomerated at 200 nm. The larger and variable sizes of Kinnow peel powder particles are visible in the TEM images.

### SEM-EDX analysis

**Figure 4:**
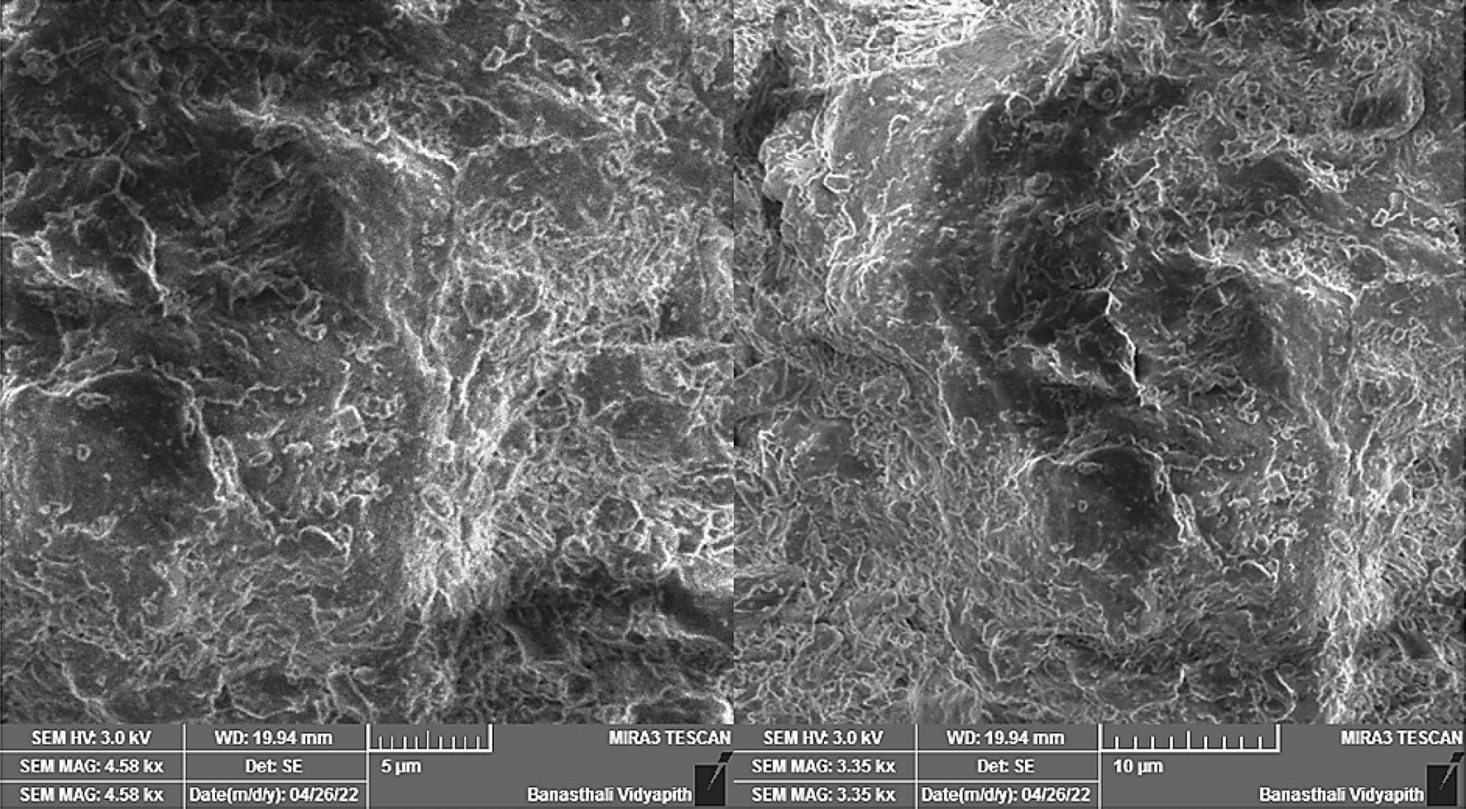
Scanning electron micrographs of the Kinnow peel powder.

**Figure 5:**
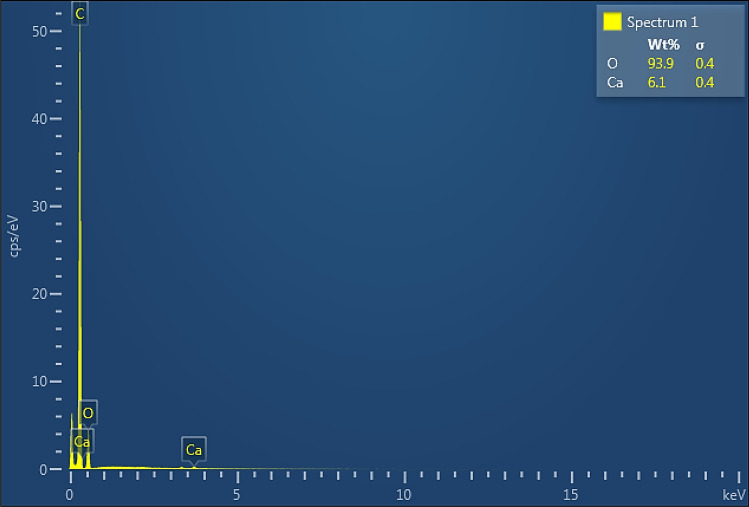
Spectrum of cracked surface of Kinnow peel powder particle based on EDX.

The scanning electron microscopy (SEM) has been used to study the morphology of Kinnow peel powder particles. Figure 4 shows the spectra obtained in SEM using EDX of the particle core. SEM spectra show the irregular particles with heterogeneous morphology. The size of the particle is 0.95 μm (calculated by imageJ). Figure 5 presents the result of EDX analysis for the cracked surface of Kinnow peel powder’s particle after autoclave. It is evident that oxygen (O) and Ca are found as major (93.9%) and minor (6.1%) elements in the sample.

### TGA analysis

**Figure 6:**
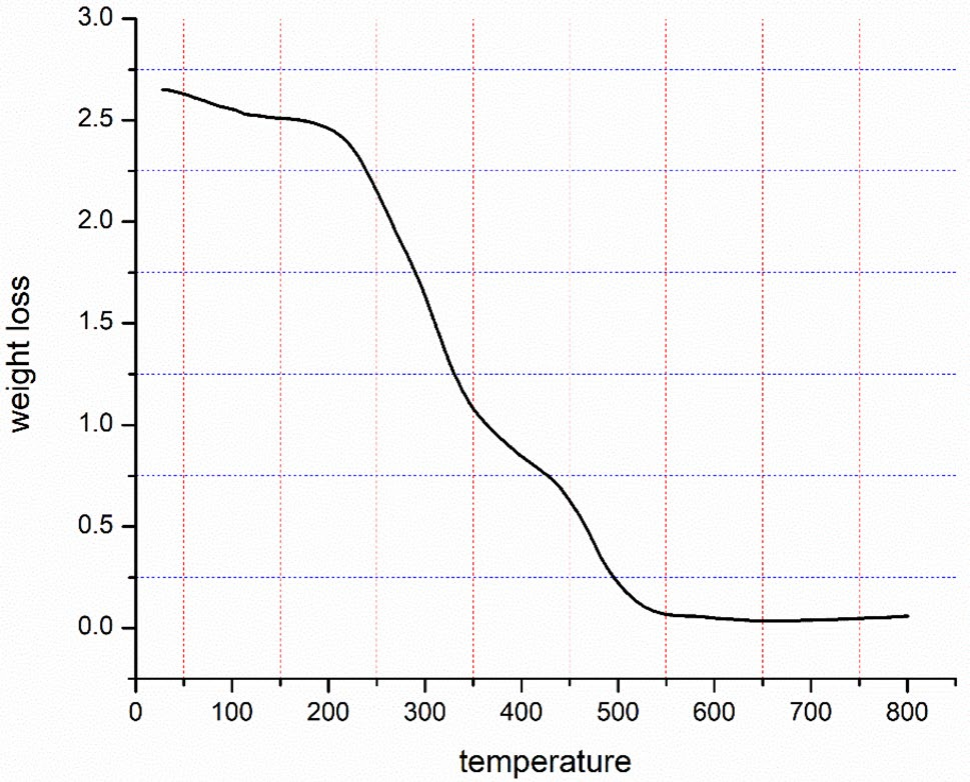
TGA analysis of Kinnow peel powder.

Thermogravimetric analysis (TGA) is used to confirm Kinnow peel powder's thermal stability throughout a temperature range of 10 °C to 800 °C. As seen in Fig. 6. The removal of the chemisorbed and physisorbed solvent over the Kinnow peel powder's surface was clearly responsible for the weight loss below 200 °C. The huge weight loss has been observed in the temperature range of 230 °C to 510 °C.

### X-ray diffraction (XRD) of Kinnow peel powder

**Figure 7:**
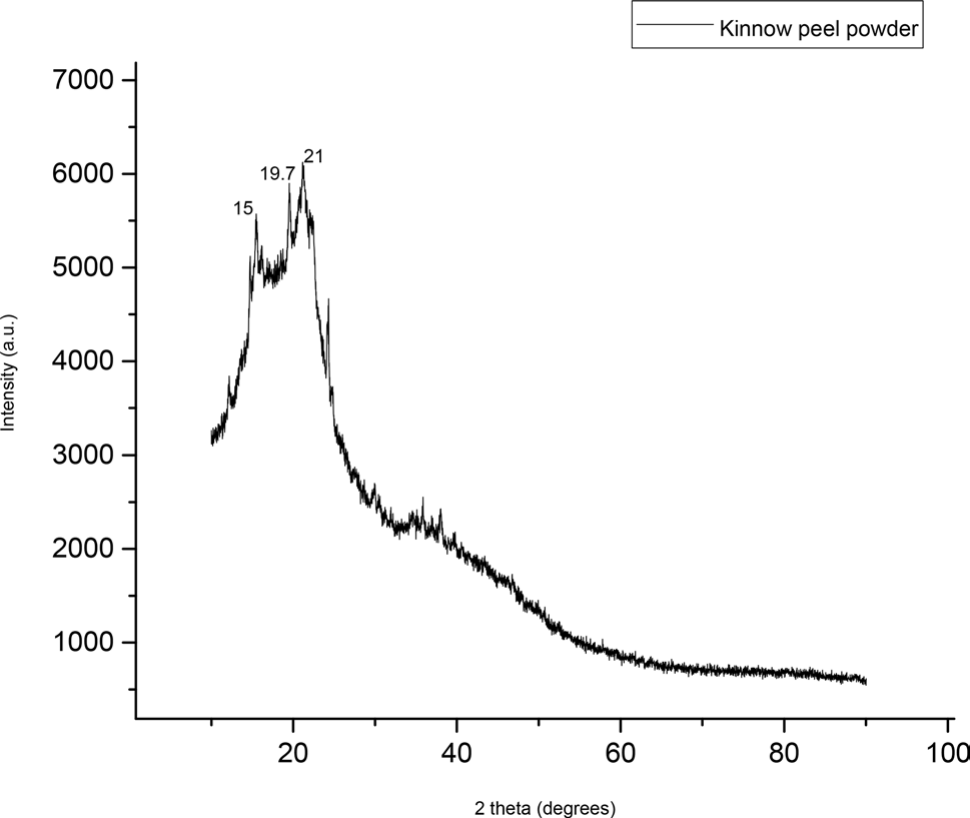
XRD data of Kinnow peel powder.

Figure 7 shows the X-ray diffraction (XRD) of Kinnow peel powder with key diffractions at 2θ = 15°, 19.7°, and 21° and it is observed with Cu Kɑ (λ = 1.5405 Å) radiation in the 2θ range from 10° to 90°.

The activity of the catalyst (Kinnow peel powder), is compared with the reported methods for Schiff base reaction and the data shown in Table 6. From the data, it is clearly shows that the high efficiency of the present work while comparing on the literature reported catalysts for the Schiff base reaction.

**Table 6:** Efficiency of the Schiff based reaction using various substrates and catalysts.

Serial no	Catalyst	Solvent	R (R-NH_2_)	R^’^ (R^’^-CHO)	Reaction condition	Time	Yield %
1	Calcined eggshell (CES)^50^	Solvent free	4-OCH_3_	4-OH	RT grinding	10–15 min	98
2	Montmorillonite K-10 clay^51^	–	H	H	MW	3 min	98
3	Acetic acid^52^	–	H	H	Grinding	2 h	89
4	Mg(ClO_4_)_2_^53^	DCE	4-NO_2_	4-OME	RT	8 h	95
5	P_2_O_5_/Al_2_O_3_^54^	–	H	H	RT string	20 min	80
6	CeCl_3_.7H_2_O^55^	Ethanol	H	2-OH	Reflux	2 h	68
7	Montmorillonite^56^	Chloroform	H	H	RT grinding	10 min	95
8	Cu/Co metal complexes^57^	Ethanol	4-NO_2_	H	Reflux	3 h	–
9	Glacial acetic acid^58^	Ethyl alcohol	4-F-2-CH_3_	H	Reflux	2 h	–
10	Hot ethanoic solution^59^	Ethanol	Substituted	(1,3-dihydrobenzoimidazole-2-ylidene)amide	Reflux	4 h	78
11	Acid catalysis^60^	Trimethyl orthoformate	H	H	Stirring	8 h	–
12	Alumina^61^	–	H	H	Stirring 20 °C	2 h	99
13	Conc. H_2_SO_4_^62^	Ethanol	–	–	Reflux	1 h	70
14	Kinnow peel powder (Present work)	Solvent free	H	H	Stirring	3 min	85

**Scheme 3:**
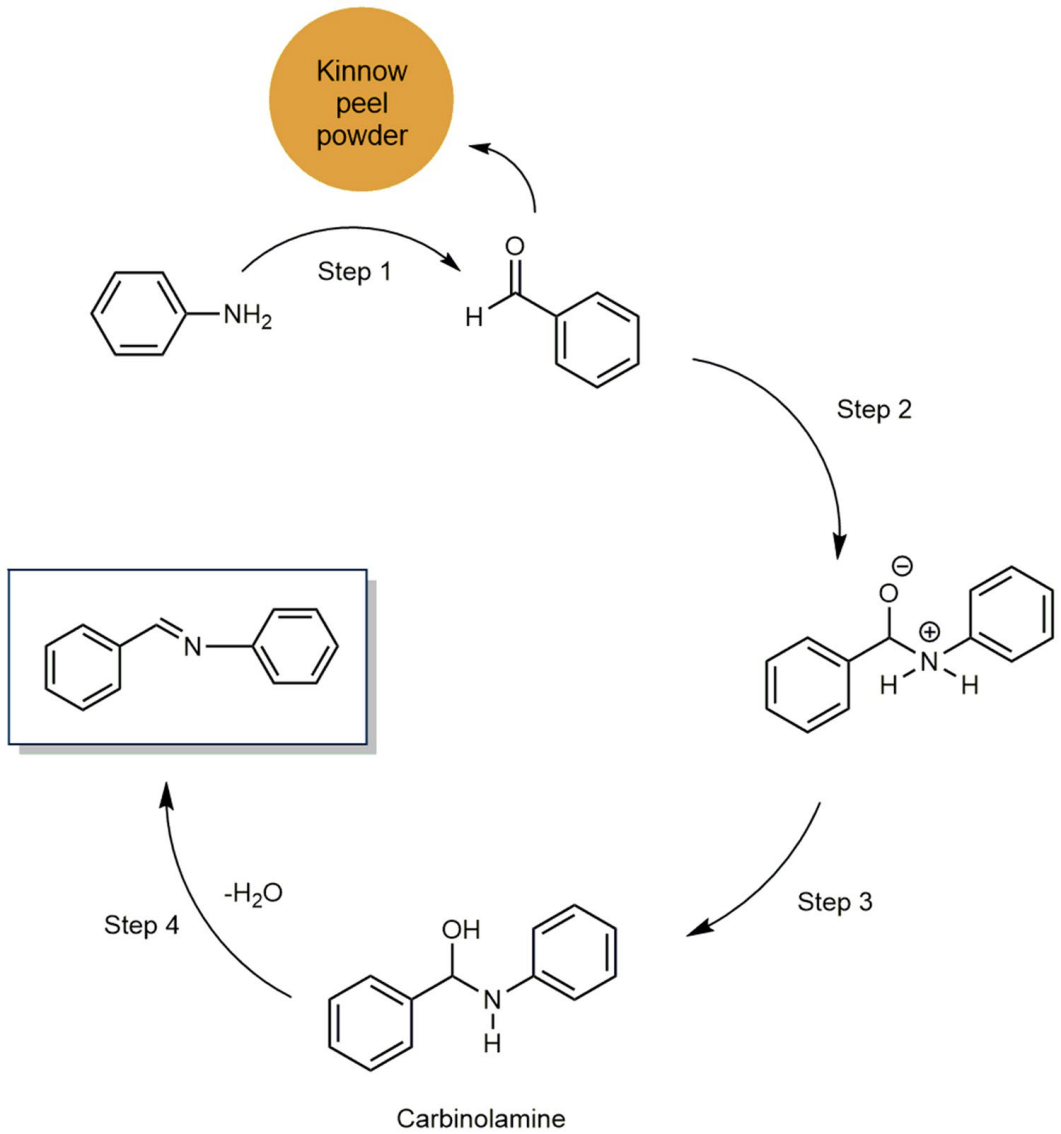
Possible Mechanism of Schiff base reaction with kinnow peel powder.

Mechanism of Schiff base formation reaction of benzaldehyde and aniline via catalyst involves four steps: (1) Reaction of aniline with benzaldehyde in the presence of kinnow peel powder (Step 1, Scheme 3), (2) formation of intermediate (Step 2, Sheme 3), (3) Formation of carbinolamine (Step 3, Scheme 3) and (4) Formation of Schiff base product (Step 4, Scheme 3)^63,64^.

## Conclusion

N-Benzalideneaniline and their derivatives are synthesized by using benzaldehyde and aniline derivatives where Kinnow peel powder has been used as a catalyst. This reaction resulted into 85% to 78% yield in neat condition. Comparisons of the yield of the six derivatives have also been done and comparative study done by ANOVA technique.
